# Elders or Old Men?

**DOI:** 10.1093/geroni/igab046.1918

**Published:** 2021-12-17

**Authors:** Thomas Cole

**Affiliations:** McGovern Medical School, Houston, Texas, United States

## Abstract

Thomas R. Cole, GSA Abstract, 3.9.2021 Elders or Old Men? My book Old Man Country is about 12 successful, respected older men who think back on their lives and current aging. When starting my research, I first questioned my own aspirations for aging: What would my aging be like? Who would I become? What would be my purpose as an old man? Although I expected that strength and resilience would be the common thread of elderhood, it was actually their vulnerabilities that defined them (accepting losses, acknowledging dependency.) More so, these vulnerabilities did not demarcate a descent but rather a continuous uphill struggle that differentiates elderhood from growing old. Ultimately, I argue that elderhood is not a life stage or a right of passage but rather an individual process to be worked through, if one so chooses.

